# Impact of Soil Nutrients on Chemical Composition and Antioxidant Activities of *Dysphania ambrosioides* Essential Oil in Southern Ecuador

**DOI:** 10.3390/plants15030373

**Published:** 2026-01-25

**Authors:** Susana Blacio, Katty Gadvay, Karen Rivas, Ana Guaman, Julio Parrales, James Calva

**Affiliations:** 1 Facultad de Ciencias Químicas y de la Salud, Universidad Técnica de Machala, Km.5 1/2 Vía Machala Pasaje, Machala 070151, Ecuador; sblacio@utmachala.edu.ec (S.B.); kgadvay@utmachala.edu.ec (K.G.); rivaskaren179@gmail.com (K.R.); jcps140398@gmail.com (J.P.); 2 Facultad de Ciencias Pecuarias y Biológicas, Universidad Técnica Estatal de Quevedo, Quevedo 120550, Ecuador; aguamang4@uteq.edu.ec; 3 Departamento de Química, Universidad Técnica Particular de Loja, Calle París s/n y Praga, Loja 110107, Ecuador

**Keywords:** essential oil, edaphic, soil, *Dysphania ambrosioides*, PCA

## Abstract

*Dysphania ambrosioides* is a widely distributed species with a traditional use in folk medicine, but it exhibits marked chemical variability that limits its standardization. This study is the first to characterize the essential oil (EO) of three Ecuadorian populations—Arenillas (ARE), Pasaje (PAS) and Piñas (PIN)—using gas chromatography–mass spectrometry/flame ionization detection (GC-MS/FID), and to correlate its composition with edaphic properties and antioxidant activity. Chemical profiles revealed three distinct chemotypes: ARE (α-terpinene 65.35%, o-cymene 24.83% and ascaridole 3.30%), PAS (α-terpinene 56.31%, o-cymene 10.09% and ascaridole 10.84%) and PIN (α-terpinene 56.89%, o-cymene 17.07% and ascaridole 7.60%). The EO yield was low (0.030–0.064% *w*/*w*), coinciding with acidic and nutrient-poor soils. On the other hand, PAS, with its neutral soil and high nitrogen, produced the highest number of oxygenated compounds. Only PAS exhibited strong ABTS radical-scavenging activity (SC_50_ = 37.99 ± 1.01 µg/mL). In contrast, ARE showed weak activity (SC_50_ = 424 ± 1.01 µg/mL), while PIN showed moderate activity (SC_50_ = 112.26 ± 1.01 µg/mL), which was correlated with its high total phenol content (298.48 mg EAG/L). The 2,2-diphenyl-1-picrylhydrazyl (DPPH) activity was low in all samples. Principal component analysis (PCA) confirmed clear separation of the chemotypes, which was linked to edaphic factors such as pH, heavy metals (Cu, Fe and Mn) and organic matter. These findings suggest that edaphic conditions may modulate the chemical composition and antioxidant activity of *D. ambrosioides*, indicating a potential approach for the sustainable selection of plant material.

## 1. Introduction

Ecuador possesses approximately 17,748 native species of vascular plants, including 1422 pteridophytes, 18 gymnosperms and 16,308 angiosperms. It has been estimated that approximately 5500 of these species are endemic, with most of them originating in the Cordillera del Cóndor [1,2]. Among these families is the Amaranthaceae, which comprises around 65 species and 900 genera distributed mainly in tropical and temperate regions and saline soils.

*Dysphania ambrosioides* is a herbaceous plant belonging to the Amaranthaceae family, native to South and Central America and widely distributed in Africa, Europe, Australia and Asian countries [3,4]. It is used for medicinal purposes and adapts easily to clayey, sandy, xerophytic and subxerophytic soils [5]. Ecologically, it is found at altitudes ranging from 0 to 2760 m above sea level [6]. It is commonly known as “epazote”, “paico”, “Jesuit tea” or “Santa María” herb. It is widely known as an effective, easy-to-administer, low-toxicity plant, perhaps considered one of the best vermifuges [7] due to its antiparasitic, antimalarial, analgesic, antimicrobial, antirheumatic and analgesic properties [8,9]. Previous studies have revealed that its essential oil contains monoterpenes such as γ-terpinene, p-cymene and 4-carene [10].

Although *D. ambrosioides* has been used traditionally and some knowledge of its composition is available, its phytochemical profile and bioactive potential still require further study to establish the relationship between its composition and specific environmental and geographical factors. The essential oil concentration and composition can be significantly influenced by edaphoclimatic factors, including altitude, climate, and the physical and chemical characteristics of the collection sites [11]. Previous studies have shown that the physical and chemical structure of the soil is one of the determining factors in the production of secondary metabolites and could also influence the composition of the volatile fraction of the same species [12], with significant variations in the major constituents and the proportions of different chemical classes, such as monoterpene hydrocarbons and oxygenated compounds, within the essential oils [13]. Sampaio et al. [14] found that the distribution of metabolites was primarily influenced by variations in certain soil nutrients, including Ca, Mg, P, K and Cu. While the chemotypic variability of *D. ambrosioides* has been documented in regions such as India, Brazil, Morocco and Taiwan, no previous study has systematically linked the composition of essential oils and their bioactivity to edaphic variables in the Andean–Amazon transition zone of southern Ecuador, a region characterized by extreme soil heterogeneity and high biodiversity.

We hypothesized that soil nutrients could influence the composition of *D. ambrosioides*. Therefore, the objectives of this study are to characterize EOs using gas chromatography coupled with mass spectrometry (GC-MS) and flame ionization detection (GC-FID) in samples collected from different locations in Ecuador. Their antioxidant capacity was evaluated for the first time in this context using the ABTS and DPPH assays, as well as total phenol content, and these parameters were correlated with the topographical, edaphic and climatic characteristics of the collection sites. For the first time, we report the existence of three distinct chemotypes in Ecuadorian populations. Each is strongly associated with site-specific edaphic signatures. This provides a geochemical framework for the rational selection of plant material in this underexplored phytogeographic hotspot. Given the chemical variability linked to edaphic conditions, this work underscores the importance of ecological context in the evaluation of *D. ambrosioides* as a potential source of bioactive volatile compounds, though further research is needed to assess its functional, agronomic or therapeutic relevance.

## 2. Results

### 2.1. Yield and Density of EOs

The yield of EOs obtained by steam distillation of *D. ambrosioides* leaves varied significantly depending on the collection site, with values of 0.064 ± 0.005% for Arenillas, 0.030 ± 0.007% for Pasaje and 0.042 ± 0.003% for Piñas.

The EO density was relatively homogeneous among the three locations, with values ranging between 0.82 and 0.83 g/mL, suggesting consistency in the overall composition of volatile compounds.

### 2.2. Chemical Composition

**Figure 1:**
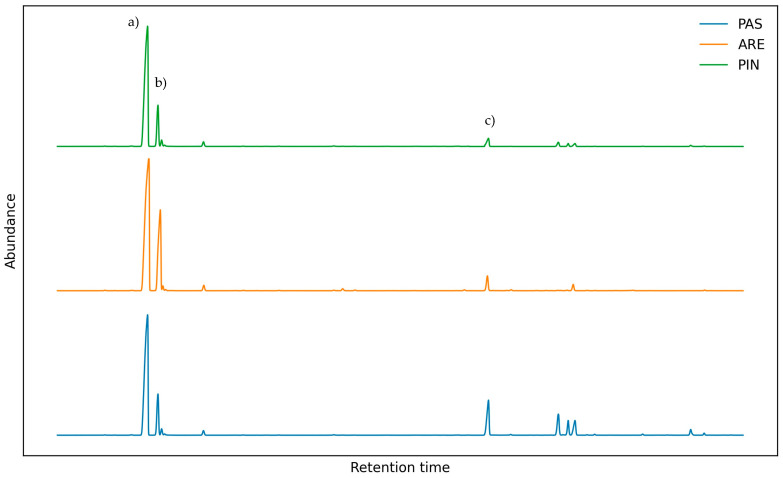
GC-MS chromatograms of *Dysphania ambrosioides* leaves of EOs from the three sectors; majority compounds: (a) α-terpinene, (b) ο-cymene and (c) ascaridole.

The EOs were analyzed using GC-MS/FID techniques with a DB5-MS column, allowing a total of 48 compounds to be identified. In ARE, 99.73% were identified; in PAS, 99.51%; and in PIN, 99.11%. The chemical composition was dominated by hydrocarbon monoterpenes (92.53% in ARE, 69.14% in PAS, 76.68% in PIN), followed by oxygenated monoterpenes (3.59% in ARE, 16.95% in PAS, 12.12% in PIN) and aldehydes (1.34% in ARE, 4.67% in PAS, 3.65% in PIN). The main components (>3%) in ARE were dominated by α-terpinene (65.35%) and o-cymene (24.83%), while ascaridole accounted for only 3.30%. In contrast, PAS had α-terpinene at 56.31%, o-cymene at 10.09% and ascaridole at 10.84%. Finally, PIN showed α-terpinene at 56.89%, o-cymene at 17.07% and ascaridole at 7.60%. These results are detailed below in Table 1, and the GC profiles are shown in Figure 1.

### 2.3. Antioxidant Activity

EOs obtained from D. ambrosioides were evaluated using ABTS and DPPH assays with Trolox as a positive control. The results are reported using the scavenging capacity (SC_50_) representing the concentration of EO required to reduce the concentration of radicals by 50%. The ABTS assay in PAS EOs (SC_50_ = 37.99 ± 1.01 µg/mL) showed antioxidant activity comparable to that of the positive control, with no statistically significant differences (*p* > 0.05). The PIN EOs (SC_50_ = 112.26 ± 1.01 µg/mL) showed moderate activity, while the ARE EOs (SC_50_ = 424 ± 1.01 µg/mL) showed insignificant activity. The DPPH assay was generally low in all EOs. However, PAS EOs (SC_50_ = 6722.42 ± 2.04 µg/mL) were significantly more active than PIN EOs (7699.58 ± 5.15 µg/mL) and ARE EOs (7578.64 ± 2.58 µg/mL) (*p* < 0.05). No significant difference was observed between ARE and PIN (Table 2).

### 2.4. Total Phenolic Content

**Figure 2:**
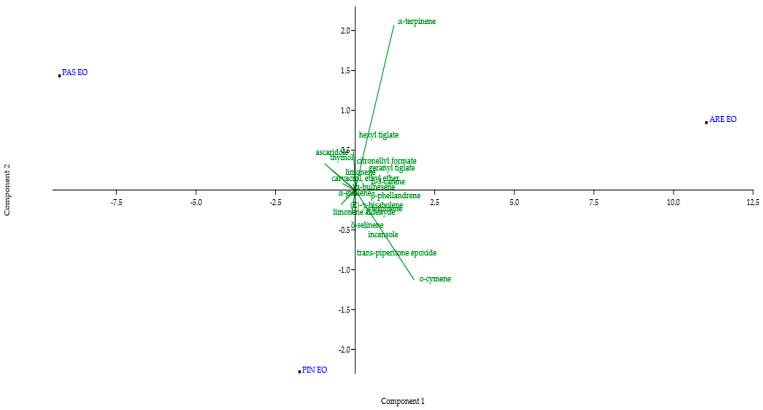
Principal component analysis (PCA) and relative compositions (%) of EOs in Arenillas (ARE), Pasaje (PAS) and Piñas (PIN). Values are expressed as % ± SD (n = 3).

The total phenolic content (TPC) in EOs, expressed in milligrams of gallic acid equivalent per liter (mg GAE/L), revealed substantial differences between populations (Table 3, Figure 2). PAS EO had the highest content (298.48 ± 8.32 mg EAG/L), followed by PIN (276.60 ± 15.58 mg EAG/L). In contrast, ARE EO had a very low TPC (23.12 ± 6.93 mg EAG/L). The coefficient of variation (CV) for all samples was low (<6%), indicating good reproducibility in measurement.

### 2.5. Principal Component Analysis (PCA)

Principal component analysis (PCA) was performed to visualize the overall chemical variability among the three populations using the percentages of the main quantified compounds (α-terpinene, o-cymene, ascaridol, limonene aldehyde, thymol and carvacrol ethyl ether) as the variables. The first principal component (PC1) accounted for 78.2% of the total variation, while the second (PC2) accounted for 14.1%. The two components together accounted for 92.3% of the variability (Figure 2).

### 2.6. Chemical Soil Analysis

Soil properties at the three collection sites showed marked differences that coincided with variations in EO yield and composition (Table 4). The soil of ARE was acidic (pH = 4.5 ± 0.10), the soil of PIN was very acidic (pH = 3.90 ± 0.00), and the soil of PAS was practically neutral (pH = 6.6 ± 0.00). ARE had a sandy-clay texture (53% sand, 22% silt, 25% clay), PAS had a distinctly clay texture (41% sand, 26% silt, 33% clay) and PIN showed a distinctly sandy texture (61% sand, 20% silt, 19% clay). The PAS soil showed the highest contents of ammoniacal nitrogen NH_4_^+^ (82.33 ppm) and phosphorus (44.67 ppm). In contrast, PIN soil had significantly higher concentrations of Cu (29.60 ppm), Fe (116.17 ppm) and Mn (19.27 ppm) compared to ARE and PAS.

The correlations between volatile compounds in essential oils and soil parameters are shown in Table 5. The Pearson correlation analysis revealed several strong positive associations (r > 0.7) between the volatile compounds in the EOs and the soil’s physicochemical properties. This indicates that, as the value of an edaphic parameter increases, so does the concentration of the volatile compound. Specifically, alpha-terpinene showed strong positive correlations with ammonium (r = 0.79461), phosphorus (r = 0.82932) and iron (r = 0.82177). Similarly, the α-cubebene compound exhibited a significant positive correlation with pH, reaching an r-value of 0.97235. Finally, the α-guaiene compound showed a strong correlation with OM (r = 0.91758) and copper (r = 0.87265). These correlations suggest that variations in the concentrations of these compounds could be significantly influenced by the levels of these nutrients and soil pH. Due to the limited number of sampling sites (n = 3), the reported Pearson correlation coefficients should be treated as descriptive only and not as evidence of statistically significant relationships. These patterns require validation using a larger spatial dataset.

## 3. Discussion

The results of this study showed the chemical variability in the essential oil of *Dysphania ambrosioides* collected in three locations in southern Ecuador, confirming the existence of three distinct chemotypes correlated with edaphic and environmental factors. α-Terpinene was the predominant metabolite at all three sites. In ARE, the chemical profile was dominated by α-terpinene (65.35%) and o-cymene (24.83%), and ascaridole accounted for 3.30%. By contrast, a more balanced composition was observed in PAS with α-terpinene (56.31%), *o*-cymene (10.09%) and ascaridole (10.84%). In PIN, an intermediate composition was recorded with α-terpinene (56.89%), o-cymene (17.07%) and ascaridole (7.60%). This pattern is consistent with previous reports in Cuba [15] and India [16], where α-terpinene was also reported as the major component (50–60%). However, the Ecuadorian samples exhibit higher levels of α-terpinene (56–65%) and *o*-cymene (10–24%), suggesting a regional trend towards the accumulation of these hydrocarbon monoterpenes. In contrast, ascaridole demonstrated significant variability (3.30–10.84%), suggesting reduced stability in its biosynthesis under local conditions. In a previous study, Hsu et al. [17] analyzed the essential oil of *D. ambrosioides* grown in Taiwan, reporting α-terpinene (30.5%), *p*-cymene (17.3%), carvacrol (16.2%) and ascaridole (15.1%) as the main constituents. Similarly, Zriouli et al. [18] identified a profile dominated by α-terpinene (53.4%), ascaridole (17.7%) and *p*-cymene (12.1%) in Morocco.

The EO yield in our study (0.030–0.064% *w*/*w*) was significantly lower than the yield reported by Jardim [19] in Brazil (0.3% *w*/*w*). This highlights the impact of intrinsic, extrinsic and environmental factors. According to research by Jabbari et al. [20], the highest EO yields are observed in soils rich in nitrogen, such as urea. Previous studies have also shown that yield changes with the seasons (1.096% in spring, 0.998% in summer, 0.774% in autumn and 0.819% in winter [21]). Additionally, high levels of certain micronutrients in the soil (e.g., Cu, Fe, Mn, K and Al) can reduce both yield and the proportion of major compounds, as demonstrated in *Thymus pulegioides* [22]. In our study, the PIN site showed high concentrations of Cu (29.60 ± 0.89 ppm), Fe 116.17 ± 5.40 ppm and Mn (19.27 ± 1.23 ppm). This coincided with an intermediate oil yield and a lower presence of the major compound, α-terpinene. While this pattern is consistent with the hypothesis that excess micronutrients may interfere with terpenoid biosynthesis [23,24], such an interpretation remains speculative in the absence of controlled nutrient-addition experiments. It is worth noting that soil texture, particularly sandy loam, has been associated with higher essential oil yields in several aromatic species, likely due to its favorable physical properties (e.g., aeration, drainage) and balanced nutrient availability, which support root development and metabolic activity [23,24].

*D. ambrosioides* EOs showed low antioxidant activity, with IC_50_ values of 2108 ± 60.15 μg/mL (ABTS) and 1597 ± 194.20 μg/mL (DPPH). There was a slight increase in ABTS activity in the PAS sample, but a poor performance in terms of DPPH. This modest activity contrasts sharply with the high antioxidant capacity reported for polar extracts of the same species. The ethyl acetate extract exhibited the highest activity in the DPPH test (IC_50_ = 25.17 μg/mL ± 0.18 μg/mL). On the other hand, in the ABTS test, the n-butanolic fraction shows the highest activity due to its low IC_50_ value of 28.19 ± 0.006 μg/mL [25]. Similarly, Ghareeb [26] observed significant activity in n-butanol and ethyl acetate extracts (with IC_50_ values of 2.98 and 16.48 mg/mL, respectively, in the DPPH test) from plants grown in Egypt. Total phenol values ranging from 23.12 ± 6.93 to 298.48 ± 8.32 mg GAE/L were obtained. The relatively low TPC values (23–298 mg GAE/L) and GC-MS data (showing only trace phenolics like thymol) suggest that the observed antioxidant activity is likely attributable to oxygenated monoterpenes rather than classical phenolic antioxidants [27]. These results contrast with those reported in extracts from Ouadja [28] of 324.8 ± 17.3 µg GAE/mg in a hydroethanolic extract and 87.69 ± 1.41 µg GAE/mg in a methanolic extract [29], and AbdulKader [30]: concentrations between 379.6 ± 0.68 and 363.6 ± 0.97 mg GAE/g. The weak antioxidant activity observed in the EOs, particularly in the DPPH assay, is consistent with their predominantly hydrocarbon composition (69–93% monoterpene hydrocarbons) and low phenolic content. Unlike polar extracts (e.g., methanolic or ethyl acetate), which concentrate phenolic compounds such as flavonoids and phenolic acids that are known to have a strong radical-scavenging capacity [31], EOs are rich in non-phenolic terpenes (e.g., α-terpinene and o-cymene) that exhibit limited electron-donating capacity. Notable ABTS activity was only observed in PAS, and total phenolics were significantly higher (298.48 mg GAE/L). This reinforces the idea that the antioxidant potential can depend largely on the type of extract, with EOs being more relevant for applications based on antimicrobial or antiparasitic properties than as primary antioxidants. The marked difference in antioxidant response between ABTS and DPPH assays reflects the distinct reaction mechanisms of these radicals. ABTS^+^• is a cationic, hydrophilic radical that can be scavenged by both hydrogen atom transfer (HAT) and single electron transfer (SET), and is more sensitive to oxygenated compounds like ascaridole and thymol. In contrast, DPPH• is a stable nitrogen-centered radical that reacts primarily via HAT and is less accessible to non-polar compounds in EOs [32]. This explains the weak DPPH activity despite moderate ABTS response in PAS oil, which contains higher levels of oxygenated monoterpenes (16.95%).

Although there are differences in altitude, temperature and humidity among the sites, the strong and significant correlations observed between specific soil parameters (e.g., pH, P and Cu) and key essential oil constituents (e.g., ascaridole and α-terpinene), as evidenced by the Pearson’s correlation matrix and PCA loadings, suggest that edaphic factors play a dominant role in shaping chemotype differentiation under the conditions studied. Nevertheless, the potential confounding influence of unmeasured climatic variables cannot be entirely ruled out. Future controlled-environment or transplant experiments are recommended. These would be used to isolate soil effects. Climatic parameters (solar radiation, temperature and relative humidity) and light variables, factors known to influence the conversion of α-terpinene to ascaridole, were not measured. In vitro studies have shown that fluorescent light promotes the formation of ascaridole, while blue LED light inhibits it [33]. Future studies are needed to explore the relationship between the quality and quantity of essential oil production in different soils and environmental conditions.

Although the Pearson correlation coefficients (r > 0.7) indicate a strong relationship between certain edaphic parameters and EO constituents, these results should be interpreted with caution, given the small number of sampling sites (n = 3). The observed correlations may be influenced by multicollinearity among soil variables (e.g., pH, OM and heavy metal availability), as these variables are often interdependent in natural ecosystems. Therefore, while the patterns are consistent and biologically plausible, they represent preliminary associations rather than definitive causal relationships. The strong positive correlation (r > 0.7) between alpha-terpinene and elements such as phosphorus (r = 0.829) and iron (r = 0.822) is particularly noteworthy. This association may be due to the important role that these micronutrients play in plant metabolic pathways. For example, iron is a cofactor in many plant enzymes involved in the electron transport chain and the synthesis of organic precursors [34]. This has an indirect effect on the mevalonate (MVA) pathway or the methyl-erythritol phosphate (MEP) pathway, which are the main pathways for the production of terpenes such as alpha-terpinene [35,36,37]. The strong positive relationship between α-cubebene and soil pH (r = 0.972) is particularly noteworthy. The availability of other nutrients could be influenced by a high pH, or soil enzyme activity could be altered, affecting the absorption of precursors and, ultimately, the gene expression that directs the synthesis of sesquiterpenes such as α-cubebene. This finding is consistent with studies showing that pH is a key environmental factor in the chemotypical variation of essential oils [38]. Finally, the strong correlations of α-guaiene with OM (r = 0.918) and copper (r = 0.873) highlight its dependence on soil chemical composition. A high OM content generally implies greater water availability and favorable soil structure, which promote vigorous plant growth and therefore greater production of secondary metabolites such as alpha-guaiene. Copper is another essential micronutrient that plays a key role in oxidation–reduction reactions, which may be related to the final stages of cyclisation in sesquiterpene synthesis [39].

The PCA biplot provides a visual summary of the chemical differences among the three populations and their association with soil variables, PC1 (78.2% variance), primarily driven by α-terpinene (positively associated with ARE) and oxygenated compounds like ascaridole and thymol (associated with PAS). Vector loadings indicate that soil pH, ammonium and phosphorus positively correlate with ascaridole, while heavy metals (Cu, Fe) are linked to hydrocarbon monoterpenes. While PCA is exploratory, these patterns support the hypothesis of edaphic modulation of chemotype expression. Soils with higher organic matter, a more neutral pH and better nutrient availability favor the greater synthesis of oxygenated monoterpenes, such as ascaridole. This compound is characteristic of this species and highly dependent on secondary metabolism, which mineral nutrition regulates [40]. In contrast, in ARE where the soil is more saline and less fertile, a different chemical profile predominates, possibly due to the plant making metabolic adjustments in response to environmental stress and nitrogen availability [41]. This relationship between soil quality and the chemical composition of the EO is consistent with previous studies showing that geographical and edaphic variation modulates the biosynthesis of volatile metabolites in *D. ambrosioides* and other aromatic species [42]. Therefore, the observed variation in chemotypes is consistent with the hypothesis that environmental factors, particularly edaphic factors, could influence the composition of essential oils. While these patterns do not confirm causality, they suggest that soil properties could be a relevant factor in future efforts to guide the sourcing or sustainable cultivation of plant material with desired phytochemical profiles.

## 4. Materials and Methods

### 4.1. Plant Material

Plant samples were collected during the same phenological stage (full flowering) in the dry season (June–July 2024) to minimize seasonal variability, in three locations (Arenillas, Pasaje and Piñas) of southern Ecuador. In Arenillas (3°32′60″ S, 80°3′36″ W) at an altitude of 15 m a.s.l., the temperature was 31 °C, and the humidity was 67%. Subsequently, in Pasaje (3°19′30″ S, 79°48′25.2″ W), the collection was performed at an elevation of 150 m a.s.l., with temperatures of 29 °C and humidity of 57%. Finally, in Piñas (3°40′51.6″ S, 79°40′51.6″ W) at an elevation of 2,400 m a.s.l., with temperatures of 27 °C and humidity of 60%, ten representative individuals were selected using a randomized stratified design, ensuring a minimum distance of 10 m between plants to avoid clonal propagation effects. Only the healthy, mature leaves from the upper third of the plant were harvested. These leaves were free from herbivory, disease or mechanical damage.

### 4.2. Extraction of Essential Oil

Essential oils were obtained by hydrodistillation using a Clevenger-type apparatus for 3 h. Samples collected in Arenillas (6000 g), Pasaje (3850 g) and Piñas (5550 g and 100 g) were placed in a 2 L round-bottom flask with 1 L of distilled water. Distillation was carried out at atmospheric pressure using a controlled heating mantle (94 ± 5 °C). The cooling water in the condenser was maintained at 10–15 °C. The essential oil was decanted, dried over anhydrous sodium sulfate (Na_2_SO_4_), and stored in amber glass vials at −20 °C until analysis.

### 4.3. Chemical Analysis

#### 4.3.1. Gas Chromatography/Mass Spectrometry (GC-MS)

The chemical analysis of the essential oils (EOs) was performed using gas chromatography–mass spectrometry (GC-MS). A Thermo Scientific™ Trace™ 1310 gas chromatograph equipped with electronic pressure control, was used and coupled to an ISQ™ 7000 single quadrupole mass spectrometry detector(Thermo Fisher Scientific, Waltham, MA, USA). Separation of the compounds was performed using a non-polar DB-5ms capillary column (30 m × 0.25 mm ID, 0.25 μm stationary phase thickness) composed of 5% phenyl-methylpolysiloxane. The oven temperature program began at 50 °C for 3 min, increasing at a rate of 3 °C/min up to 230 °C. Injection was performed in split mode (50:1) at 250 °C with an injection volume of 1 µL. The DB-5MS column reached a maximum temperature of 350 °C. The carrier gas was nitrogen, and operation involved a constant flow rate of 1 mL/min with an initial pressure of 6.49 psi and an average linear velocity of 35 cm/s. The samples were injected in triplicate (n = 3) to ensure reproducibility and analytical representativeness.

The compound identification was performed by comparing the experimental mass spectra with the NIST 21 [43] and Adams [44] databases and calculating the Linear Retention Index (LRI) according to the Van den Dool and Kratz [45] method, using a mixture of n-alkanes (C_10_–C_25_), which were injected under identical chromatographic conditions. Components were considered tentatively identified when both spectral match (similarity ≥85%) and LRI deviation (≤±20 units) criteria were met. Although confirmation with authentic standards would further increase the confidence in compound identification, the determination of retention indices and their comparison with available reference databases, together with mass spectral data, represents the standard and widely accepted approach for compound identification in the phytochemical analysis of complex essential oils [46,47].

#### 4.3.2. Gas Chromatography/Flame Ionization Detector (GC-FID)

GC-FID was used to quantify the EO components of *D. ambrosioides*, using the same chromatographic conditions and column described above. The relative percentage of each compound was calculated from the area of the corresponding peak in the chromatogram, normalized to the total area of all the detected peaks. No correction factors were applied.

### 4.4. Antioxidant Capacity

#### 4.4.1. DPPH Radical-Scavenging Assay

The DPPH radical-scavenging assay was adapted from that described by Thaipong et al. [48] and previously optimized by Calva et al. [49]. A stock solution of DPPH in methanol (625 µM) was prepared and diluted to an absorbance of 1.1 ± 0.02 at 515 nm to obtain the working solution. Spectrophotometric readings were performed using an EPOCH microplate reader (Epoch 2, BioTek, VT, USA) at 515 nm and 96-well plates. A 270 µL volume of the DPPH working solution was mixed with 30 µL of the sample, and the mixture was incubated for 60 min at room temperature in the dark. The blank consisted of 270 µL of DPPH and 30 µL of methanol. Trolox was used as a positive control. The essential oil samples were dissolved in methanol at a concentration of 10 mg/mL and subjected to three serial dilutions (1:10). Each assay was performed in triplicate. Statistical analyses were performed using GraphPad Prism V.8.0.1 software with a non-linear regression model (log inhibitor vs. normalized response—dependent variable). The response is expressed as the half-scavenging capacity (SC_50_), indicating the amount of analyte needed to reduce the presence of the radical in the assay by 50%.

#### 4.4.2. ABTS Radical-Scavenging Assay

The antioxidant capacity assay using the cationic radical ABTS^•+^ was adapted from the methodologies described by Thaipong et al. [48] and Arnao et al. [50]. The ABTS^•+^ radical was generated by the equimolar reaction of an aqueous solution of ABTS (7.4 mM) with potassium persulfate (2.6 mM). The mixture was then incubated for 24 h at room temperature in the dark to ensure complete radical formation. The working solution was prepared by diluting the ABTS stock solution in methanol to give an initial absorbance of 1.1 ± 0.02 at 734 nm. Measurements were performed using an EPOCH microplate reader (BioTek Instruments) and 96-well plates. Following the same design as for the DPPH system, 270 µL of the ABTS working solution was mixed with 30 µL of different essential oil (EO) concentrations and incubated for 60 min in the dark at room temperature. Methanol was used as a blank instead of a sample, and Trolox was used as a positive control. Statistical analyses for ABTS were performed using the same non-linear regression model in GraphPad Prism V.8.0.1 software, following the same procedure as for DPPH.

### 4.5. Total Phenol Content Analysis

Quantification of the total phenolic compounds was performed according to the method described by Thaipong et al. [48], adapted for implementation in a 96-well microplate system with a final reaction volume of 300 µL. In each well, 255 µL of ultrapure water, 15 µL of Folin–Ciocalteu reagent (diluted to 0.25 N) and 30 µL of 1 N sodium carbonate solution were mixed. This mixture was then carefully homogenized and incubated for two hours at 22 °C in the dark in an Epoch 2 microplate reader (Epoch 2, BioTek, VT, USA). Each sample was read in triplicate at an absorbance of 756 nm.

### 4.6. Chemical Analysis of Soils

Soil samples were collected simultaneously with plant material at each site. Five subsamples (at a depth of 0–20 cm) were taken in a zigzag pattern around each sampling point for plants, and these were combined to form a single composite sample per location (n = 3 composite samples in total), using a Riverside-type auger at the sites described above. The samples were not subjected to any pre-treatment. To determine the texture and textural class of the soil, the hydrometer method [51] was used to report the percentages of sand, silt and clay. The pH was calculated using a pH tester (iNOA^®^ pH 7310, WTW, Munich, Germany), and the electrical conductivity (EC) was measured using a conductivity meter (iNOA^®^ Cond 7310, WTW, Munich, Germany) in a soil/water suspension (1:2.5). The organic matter content was estimated using the loss on ignition method at 600 °C for two hours, applying the equation proposed by Schulte and Hopkins (1996) and cited by Eyherabide et al. [52]. All analyses were performed in triplicate.

#### Nutrients Analyzed

The samples were analyzed using a digestion method, with 0.5 g of sample and 10 mL of 65% analytical-grade HNO_3_ (maximum 0.005 ppm Hg) from Merck (Darmstadt, Germany). Digestion was performed in a CEM Mars 6 microwave system (manufactured by CEM Corp., Matthews, NC, USA) for 40 min. The final volume was adjusted to 50 mL with double-distilled water [53], after which the samples were measured using an Atomic Absorption Analyst 400 (PerkinElmer, Waltham, MA, USA). The ammonium (NH_4_), phosphorus (P), zinc (Zn), copper (Cu), iron (Fe), manganese (Mn), potassium (K), calcium (Ca) and magnesium (Mg) concentrations were determined for each parameter using certified Merck standards. The results were statistically analyzed using linear regression and two independent measures to determine the analytical accuracy and precision of the method.

### 4.7. Statistical Analysis

All analytical determinations were performed in triplicate. Data normality was assessed using the Shapiro–Wilk test, and homogeneity of variance by Levene’s test [54]. One-way ANOVA followed by Tukey’s post hoc test (α = 0.05) was used to compare means among sites for soil properties, essential oil yield and antioxidant activity. Pearson’s correlation coefficients were calculated to assess the relationships between soil parameters and major volatile compounds. PCA was performed on standardized data (mean-centered and unit-variance scaled) using XLSTAT 2023 or GraphPad Prism v8.0.1. Statistical significance was set at *p* < 0.05.

## 5. Conclusions

This study showed that the chemical composition and antioxidant activity of essential oil from *D. ambrosioides* in southern Ecuador may mainly be affected by soil factors. Although all populations share the same major compounds (α-terpinene, o-cymene, ascaridole), significant quantitative differences (*p* < 0.05, ANOVA) and distinct associations with soil parameters justify the identification of three quantitative chemotypes: (i) a hydrocarbon-dominant type (ARE), (ii) an oxygenated-monoterpene-enriched type (PAS) and (iii) an intermediate profile (PIN). The PAS chemotype, which was associated with a neutral pH level, high nitrogen content and high phosphorus content, produced the highest levels of ascaridol and exhibited ABTS antioxidant activity that was comparable to that of Trolox. This is consistent with its high total phenolic content (298.48 mg EAG/L). In contrast, the ARE chemotype, which is found in acidic, low-fertility soil, produced high levels of α-terpinene but minimal ascaridol. The PIN soil showed toxic levels of copper (Cu), iron (Fe) and manganese (Mn), and inhibited ascaridol biosynthesis despite intermediate terpene levels. Although the patterns observed suggest a possible relationship between soil properties and essential oil composition, the correlative nature of this study prevents definitive conclusions from being drawn. To achieve greater precision and validate the observed relationships between soil properties and chemotypes, future studies should consider more variables.

## Figures and Tables

**Table 1:** Chemical composition of EO from *Dysphania ambrosioides* determined by GC/MS.

N°	Compound	LRI ^a^	LRI ^b^	ARE	PAS	PIN
				% ± SD		
1	myrcene	991	990	0.10 ± 0.02	0.08 ± 0.02	0.09 ± 0.01
2	δ-3-carene	1011	1011	0.11 ± 0.01	0.11 ± 0.03	0.09 ± 0.01
3	α-terpinene	1023	1017	65.35 ± 0.72	56.31 ± 1.22	56.89 ± 0.76
4	ο-cymene	1030	1026	24.83 ± 0.24	10.09 ± 0.26	17.07 ± 0.14
5	limonene	1033	1029	0.75 ± 0.04	1.26 ± 0.06	0.97 ± 0.07
6	β-phellandrene	1035	1029	0.21 ± 0.02	0.20 ± 0.04	0.24 ± 0.01
7	γ-terpinene	1061	1059	1.10 ± 0.04	1.08 ± 0.03	1.18 ± 0.13
8	terpinolene	1089	1088	0.08 ± 0.01	--	0.15 ± 0.01
9	n-nonanal	1113	1100	0.07 ± 0.01	--	--
10	cis-pinene hydrate	1148	1143	--	0.15 ± 0.04	0.09 ± 0.01
11	unidentified	1148	--	0.13 ± 0.01	--	0.75 ± 0.01
12	2-acetyl-5-methyl-furan	1154	1037	0.44 ± 0.03	--	0.34 ± 0.04
13	unidentified	1162	n.d.	0.15 ± 0.01	--	0.15 ± 0.01
14	linalool formate	1237	1216	0.18 ± 0.01	--	--
15	ascaridole	1253	1237	3.30 ± 0.07	10.84 ± 0.53	7.60 ± 0.10
16	trans-piperitone epoxide	1267	1256	0.07 ± 0.02	--	0.85 ± 0.02
17	citronellyl formate	1270	1273	0.20 ± 0.04	0.15 ± 0.03	--
18	menthyl acetate	1296	1295	0.05 ± 0.01	--	0.06 ± 0.01
19	α-terpinen-7-al	1300	1285	0.03 ± 0.01	--	0.07 ± 0.01
20	thymol	1306	1290	0.08 ± 0.01	5.84 ± 0.11	3.37 ± 0.10
21	trans-ascaridol glycol	1310	1273	0.12 ± 0.04	0.12 ± 0.03	0.14 ± 0.01
22	carvacrol, ethyl ether	1315	1298	0.08 ± 0.02	3.16 ± 0.10	1.91 ± 0.07
23	limonene aldehyde	1320	1328	1.27 ± 0.04	4.67 ± 0.05	3.65 ± 0.07
24	1,8-octanediol	1331	1341	0.08 ± 0.01	0.09 ± 0.03	0.09 ± 0.01
25	hexyl tiglate	1338	1332	0.35 ± 0.03	0.81 ± 0.07	--
26	longicyclene	1370	1374	0.19 ± 0.02	--	--
27	α-cubebene	1379	1351	--	0.24 ± 0.05	0.33 ± 0.00
28	α-guaiene	1423	1439	--	1.41 ± 0.01	0.97 ± 0.05
29	unidentified	1436	n.d.	--	0.38 ± 0.06	--
30	linalool isovalerate	1461	1468	--	0.07 ± 0.01	0.06 ± 0.01
31	β-vetispirene	1476	1488	--	0.08 ± 0.01	--
32	δ-selinene	1492	1492	--	0.71 ± 0.07	0.84 ± 0.07
33	α-bulnesene	1496	1509	--	0.18 ± 0.05	0.09 ± 0.01
34	valencene	1498	1496	--	0.04 ± 0.02	0.02 ± 0.01
35	bicyclogermacrene	1502	1500	0.04 ± 0.01	0.24 ± 0.04	0.08 ± 0.01
36	β-bisabolene	1513	1505	--	0.10 ± 0.03	--
37	selina-3,7(11)-diene	1529	1546	--	0.05 ± 0.02	0.01 ± 0.01
38	(*E*)-γ-bisabolene	1534	1531	--	0.47 ± 0.04	0.36 ± 0.00
39	selin-11-en-4-α-ol	1640	1659	0.05 ± 0.01	0.07 ± 0.01	0.03 ± 0.01
40	geranyl tiglate	1674	1696	0.15 ± 0.02	0.14 ± 0.03	--
41	(2*Z*,6*Z*)-farnesal	1684	1684	0.20 ± 0.02	0.50 ± 0.07	0.12 ± 0.02
42	epi-α-bisabolol	1699	1684	--	0.09 ± 0.03	0.05 ± 0.01
43	geranyl tiglate	1714	1696	0.02 ± 0.01	--	0.09 ± 0.01
44	n-octadecane	1801	1800	--	0.07 ± 0.01	0.08 ± 0.01
45	unidentified	1811	--	--	0.12 ± 0.03	--
46	(*Z*,*E*)-geranyl linalool	1993	1998	--	0.09 ± 0.01	0.16 ± 0.01
47	n-octadecanol	2097	2077	0.10 ± 0.03	--	0.22 ± 0.01
48	incensole	2119	2159	0.14 ± 0.05	--	0.76 ± 0.01
	Total identified (%)			99.73	99.51	99.11
	Monoterpene hydrocarbons (%)			92.53	69.14	76.68
	Oxygenated monoterpenes (%)			3.59	16.95	12.12
	Aldehydes (%)			1.34	4.67	3.65
	Esters (%)			0.85	4.12	1.97
	Oxygenated sesquiterpenes (%)			0.43	0.86	0.35
	Sesquiterpene hydrocarbons (%)			0.23	3.52	2.69
	Oxygenated diterpenes			0.14	0	0.76
	Alcohols (%)			0.1	0	0.22
	Terpenes (%)			0	0.09	0.16
	Alkanes (%)			0	0.07	0.08
	Others (%)			0.52	0.09	0.43

^a^ LRI: calculated retention index; ^b^ LRI: retention index according to Adams literature and the NIST platform; ARE: Arenillas; PAS: Pasaje; PIN: Piñas; % ± SD: mean ± standard deviation (n = 3).

**Table 2:** Radical-scavenging capacity of *Dysphania ambrosioides* EOs in Arenillas (ARE), Pasaje (PAS) and Piñas (PIN). Values are expressed as % ± SD (n = 3) (*p* < 0.05).

Sample	ABTS	DPPH
	SC_50_ (µg/mL—µM *) ± SD	
ARE EO	424 ± 1.01	7578.64 ± 2.58
PAS EO	37.99 ± 1.01	6722.42 ± 2.04
PIN EO	112.26 ± 1.01	7699.58 ± 5.15
Trolox *	29.09 ± 1.05	

* Positive control.

**Table 3:** Total phenolic content of *Dysphania ambrosioides* EOs in Arenillas (ARE), Pasaje (PAS) and Piñas (PIN). Values are expressed as % ± SD (n = 3) (*p* < 0.05).

Sample	Phenolic Content mg EAG/L	CV (%)
ARE EO	23.12 ± 6.93	3.1
PAS EO	298.48 ± 8.32	2.78
PIN EO	276.60 ± 15.58	5.63

CV = Coefficient of variation; milligrams gallic acid equivalent per liter.

**Table 4:** Chemical analysis of the soils in Arenillas (ARE), Pasaje (PAS) and Piñas (PIN). Values are expressed as % ± SD (n = 3) (*p* < 0.05).

Parameter	Unit of Measurement	ARE	PAS	PIN
Potential of Hydrogen (pH)		4.5 ± 0.10	6.6 ± 0.10	3.90 ± 0.10
E.C.	dS/m	0.44 ± 0.03	0.35 ± 0.04	0.01 ± 0.01
O.M. (%)	(%)	1.71 ± 0.07	1.36 ± 0.02	1.64 ± 0.06
Ammonium (NH4)	ppm	32.33 ± 2.52	82.33 ± 2.08	14.33 ± 1.53
Phosphorus (P)		20.00 ± 2.00	44.67 ± 5.03	8.67 ± 1.15
Zinc (Zn)		5.57 ± 1.34	5.50 ± 1.95	9.97 ± 1.22
Copper (Cu)		6.77 ± 1.27	4.43 ± 0.47	29.60 ± 0.89
Iron (Fe)		93.17 ± 2.76	40.50 ± 1.90	116.17 ± 5.40
Manganese (Mn)		12.47 ± 1.82	11.20 ± 2.27	19.27 ± 1.23
Potassium (K)	meq/100 g	0.52 ± 0.12	0.29 ± 0.02	0.37 ± 0.02
Calcium (Ca)		17.47 ± 0.50	17.07 ± 0.29	15.54 ± 0.99
Magnesium (Mg)		6.90 ± 0.41	5.71 ± 0.24	2.79 ± 0.21

E.C.: electrical conductivity of soil; O.M.: organic matter; %: percentage; dS/m: decimicrometers per meter; ppm: parts per million; meq/100 g: milliequivalents per 100 g of soil.

**Table 5:** Pearson correlation matrix between volatile compounds in essential oil and soil edaphic parameters.

	pH	E.C	O.M. (%)	Ammonium (NH_4_)	Phosphorus (P)	Zinc (Zn)	Copper (Cu)	Iron (Fe)	Manganese (Mn)	Potassium (K)	Calcium (Ca)	Magnesium (Mg)
δ-3-carene	0.53093	0.12716	0.7877	0.50222	0.46751	0.0087014	0.053627	0.47506	0.093496	0.88927	0.12581	0.18156
α-terpinene	0.7659	0.57601	0.50912	0.79461	0.82932	0.71187	0.7568	0.82177	0.79667	0.18609	0.57736	0.52161
ο-cymene	0.48832	0.85359	0.23154	0.51702	0.55174	0.98946	0.96562	0.54418	0.92575	0.091489	0.85494	0.79919
limonene	0.41873	0.92318	0.16195	0.44743	0.48215	0.94096	0.89603	0.4746	0.85616	0.16108	0.92453	0.86878
β-phellandrene	0.37651	0.28158	0.63328	0.3478	0.31309	0.14572	0.10079	0.32064	0.060924	0.95631	0.28023	0.33598
γ-terpinene	0.41371	0.24439	0.67048	0.385	0.35029	0.10852	0.063595	0.35784	0.023726	0.99351	0.24303	0.29879
terpinolene	0.1731	0.48499	0.42988	0.1444	0.10968	0.34912	0.3042	0.11724	0.26433	0.75291	0.48364	0.53939
cis-pinene hydrate	0.54226	0.79965	0.28548	0.57096	0.60568	0.93551	0.98044	0.59813	0.97969	0.037547	0.801	0.74525
2-acetyl-5-methyl-furan	0.27485	0.93294	0.01807	0.30355	0.33827	0.79707	0.75215	0.33071	0.71228	0.30496	0.93159	0.98734
linalool formate	0.8024	0.5395	0.54563	0.83111	0.86582	0.67537	0.72029	0.85827	0.76016	0.2226	0.54086	0.4851
ascaridole	0.52063	0.82128	0.26385	0.54934	0.58405	0.95714	0.99793	0.5765	0.95806	0.059175	0.82263	0.76688
trans-piperitone epoxide	0.48366	0.17443	0.74044	0.45496	0.42024	0.038565	0.006361	0.42779	0.04623	0.93653	0.17308	0.22883
citronellyl formate	0.68535	0.02726	0.94213	0.65664	0.62193	0.16312	0.20805	0.62948	0.24792	0.73485	0.02861	0.027143
thymol	0.52121	0.8207	0.26443	0.54991	0.58463	0.95657	0.99851	0.57707	0.95864	0.058598	0.82205	0.7663
trans-ascaridol glycol	0.53093	0.12716	0.7877	0.50222	0.46751	0.0087014	0.053627	0.47506	0.093496	0.88927	0.12581	0.18156
carvacrol, ethyl ether	0.53801	0.80389	0.28124	0.56672	0.60143	0.93976	0.98468	0.59388	0.97545	0.04179	0.80525	0.74949
limonene aldehyde	0.61356	0.72835	0.35678	0.64227	0.67698	0.86421	0.90914	0.66943	0.94901	0.033755	0.7297	0.67395
hexyl tiglate	0.14778	0.51031	0.40456	0.11908	0.084363	0.37444	0.32952	0.09192	0.28965	0.72759	0.50896	0.56471
longicyclene	0.8024	0.5395	0.54563	0.83111	0.86582	0.67537	0.72029	0.85827	0.76016	0.2226	0.54086	0.4851
α-cubebene	0.97235	0.36955	0.71558	0.99894	0.96423	0.50542	0.55035	0.97178	0.59021	0.39255	0.37091	0.31515
α-guaiene	0.60512	0.73679	0.34834	0.63383	0.66854	0.87265	0.91758	0.66099	0.95745	0.025315	0.73814	0.68239
unidentified	0.13574	0.79383	0.12104	0.16444	0.19916	0.65797	0.61304	0.19161	0.57317	0.44407	0.79248	0.84823
δ-selinene	0.89424	0.44766	0.63747	0.92295	0.95766	0.58353	0.62846	0.95011	0.66832	0.31444	0.44902	0.39326
α-bulnesene	0.46907	0.87284	0.2123	0.49778	0.53249	0.9913	0.94637	0.52494	0.9065	0.11073	0.87419	0.81844
valencene	0.46907	0.87284	0.2123	0.49778	0.53249	0.9913	0.94637	0.52494	0.9065	0.11073	0.87419	0.81844
bicyclogermacrene	0.25677	0.91487	9.00E-06	0.28548	0.3202	0.779	0.73408	0.31264	0.69421	0.32303	0.91352	0.96927
(*E*)-γ-bisabolene	0.65876	0.68315	0.40198	0.68746	0.72218	0.81901	0.86394	0.71463	0.90381	0.078953	0.6845	0.62875
geranyl tiglate	0.56891	0.08919	0.82568	0.5402	0.50549	0.046679	0.091605	0.51304	0.13147	0.85129	0.087833	0.14359
(2*Z*,6*Z*)-farnesal	0.007765	0.66586	0.24901	0.036472	0.071185	0.52999	0.48507	0.06363	0.4452	0.57204	0.66451	0.72026
(*Z*,*E*)-geranyl linalool	0.91013	0.25203	0.8331	0.88142	0.84671	0.3879	0.43283	0.85426	0.47269	0.51007	0.25339	0.19763
n-octadecanol	0.23098	0.42711	0.48775	0.20227	0.16756	0.29125	0.24632	0.17511	0.20645	0.81078	0.42576	0.48151
incensole	0.4202	0.2379	0.67697	0.39149	0.35678	0.10203	0.057106	0.36433	0.017237	1	0.23654	0.2923

The shading indicates the most important correlations in this Pearson method.

## Data Availability

The original contributions presented in this study are included in the article. The raw data supporting the conclusions of this article will be made available by the corresponding author.
